# Innovative ohmic-sous vide cooking technique for enhancing physicochemical, textural, and sensory attributes of Arabian camel (*Camelus dromedarius*) meat

**DOI:** 10.1371/journal.pone.0359223

**Published:** 2026-11-03

**Authors:** Bandar Alfaifi, Ali I. Hobani, Gamaleldin M. Suliman, Mansour N. Ibrahim, Ronnel B. Fulleros, Moath B. Othman

**Affiliations:** 1 Department of Agricultural Engineering, College of Food and Agriculture Sciences, King Saud University, Riyadh, Saudi Arabia; 2 Department of Animal Production, College of Food and Agricultural Sciences, King Saud University, Riyadh, Saudi Arabia; Yantai Institute of Technology, CHINA

## Abstract

This study evaluated the effects of ohmic-sous vide (OS) and conventional sous vide (SV) cooking on the physicochemical, color, textural, and sensory properties of Arabian camel (*Camelus dromedarius*) meat. Meat samples were cooked at 70, 80, and 90 °C for 30, 60, 90, and 120 min using a 2 × 3 × 4 factorial design. Compared with SV cooking, OS cooking produced significantly higher pH, cooking loss, water activity, yellowness (b*), total color change (ΔE), hue angle, and chroma values, whereas lightness (L*), redness (a*), and shear force were significantly lower (p < 0.001). Increasing cooking temperature and time generally increased pH, cooking loss, yellowness, total color change, hue angle, and chroma, while decreasing lightness, redness, water activity, and shear force. Sensory evaluation showed that OS cooking significantly improved overall acceptability (p < 0.001) and tenderness (p = 0.01) compared with SV cooking. In contrast, cooking method did not significantly affect flavor or juiciness (p > 0.05). Increasing cooking temperature and time improved overall acceptability, tenderness, and flavor but progressively reduced juiciness. Meat cooked at 90 °C for 120 min received the highest overall acceptability, tenderness, and flavor scores, although this treatment also resulted in the lowest juiciness. In conclusion, OS cooking improved tenderness and sensory acceptability compared with conventional SV cooking; however, these benefits were accompanied by greater cooking loss and changes in meat color. These findings highlight the potential of OS cooking for improving camel-meat tenderness while emphasizing the need to optimize processing conditions to balance tenderness, moisture retention, color, and sensory quality.

## 1. Introduction

Camel meat is an important source of red meat in the Kingdom of Saudi Arabia, contributing approximately 2.8 kg/person/year to per capita meat consumption and serving as a valuable source of dietary protein in several Middle Eastern countries. Chad followed by Somalia possess the largest populations of Arabian one-humped camels worldwide [1]. In Saudi Arabia, the camel population was estimated at approximately 2.24 million head in 2024, representing nearly 5% of the global camel population [2].

Compared with other red meats, camel meat possesses several desirable nutritional attributes. It contains higher moisture, essential amino acids, minerals, and vitamins while being naturally lean with relatively low intramuscular fat and cholesterol levels [3]. Camel meat is composed of approximately 78% water, 19% protein, 3% fat, and 1.2% ash, and contains lower total fat, cholesterol, and ash than beef and other common red meats [4]. For example, camel meat contains only 1.54% fat compared with 2.74% in beef, while its cholesterol content (57.56 ± 4.66 mg/100 g) is considerably lower than that of beef (74.50 ± 6.73 mg/100 g) [5]. These nutritional characteristics make camel meat an attractive alternative protein source for health-conscious consumers.

Although camel meat is traditionally prepared in Saudi Arabia and other Middle Eastern countries using slow roasting, stewing, braising, grilling, boiling, and pressure cooking, recent advances in thermal processing have introduced innovative alternatives that may further improve meat quality. Sous vide [6,7] and ohmic heating [8–10] are among the processes that have attracted considerable attention because of their potential to enhance cooking efficiency and product quality.

Sous vide (SV) cooking, also known as under vacuum cooking, involves heating vacuum-sealed food at precisely controlled temperatures for predetermined times [6]. The technique has been widely reported to improve moisture retention, tenderness, flavor, aroma, color uniformity, and texture while reducing microbial populations [7,11]. However, its relatively long processing times contribute to high energy consumption, limiting its industrial efficiency [12,13]. Previous studies have demonstrated that SV cooking conditions markedly influence meat quality. Hobani, Othman [6] reported that cooking method, temperature, and time significantly affected the physicochemical and sensory properties of camel meat. Compared with electric-oven cooking, SV cooking improved tenderness, flavor, overall acceptability, density, textural uniformity, and color preservation, indicating its suitability for camel meat processing when moisture loss is properly controlled. Similarly, Othman, Alfaifi [14] compared conventional SV with ohmic-sous vide (OS) cooking of camel meat at 70–90°C for 30–120 min and found that OS substantially reduced preheating time and energy consumption while improving energy efficiency. However, increasing cooking temperature and duration decreased the thermophysical properties, density, and cooking yield under both cooking methods. Comparable findings were reported by Sun, Rasmussen [15], who observed that non-HPP beef steaks cooked SV at 55°C for 180 min achieved the highest tenderness, juiciness, and overall acceptability. Although HPP-treated steaks exhibited higher lightness and yellowness without affecting redness, longer SV cooking enhanced tenderness, whereas higher pressure combined with shorter cooking times reduced sensory quality. These findings suggest that optimizing HPP-SV processing can improve microbial safety and processing efficiency while maintaining acceptable product quality. Likewise, Li [16] concluded that SV cooking generally enhances meat tenderness and juiciness while reducing shear force and toughness, although the magnitude of these effects depends on meat type and processing conditions. Extended cooking times further increased tenderness but also promoted moisture loss, whereas higher cooking temperatures adversely affected juiciness. Collectively, these studies highlight the advantages of SV cooking in providing precise temperature control while improving the overall quality of meat products.

Ohmic heating is a modern thermal processing technology in which heat is generated internally by passing an electric current directly through the food [10 14]. Unlike conventional water-based cooking methods, where heat is transferred from an external medium [16–18], ohmic heating produces rapid and volumetric heating without requiring a heat transfer medium [19,20]. Previous studies have demonstrated reduced cooking losses (27.7%) compared with steam cooking (37.9%) [21], lower energy consumption [19], improved retention of nutritional quality and color, and enhanced microbial inactivation resulting from rapid volumetric heating under appropriate processing conditions [8,9,22–24]. Moreover, two-stage ohmic treatment has been shown to further enhance microbial inactivation and extend the shelf life of cooked meat products [22,25,26]. However, these benefits depend on achieving a sufficiently uniform electric field and temperature distribution. Variations in electrical conductivity among food components may create localized hot and cold spots, leading to uneven heating that can compromise both product quality and microbial safety if not properly controlled. In addition, excessive electrical heating may alter protein structure and slightly affect the final product color [8,15].

Camel meat is generally characterized by greater chewiness than other red meats [27], making optimization of cooking methods particularly important. The physicochemical and textural properties of camel meat, including pH, cooking loss, water activity, color, and shear force, are strongly influenced by cooking temperature, processing time, and cooking method [28]. These changes directly influence palatability by affecting tenderness, juiciness, flavor, and overall consumer acceptance. Previous studies have shown that improved tenderness and moisture retention are associated with greater consumer preference, whereas excessive cooking loss and reduced water-holding capacity negatively affect eating quality [27]. During cooking, structural modifications including protein denaturation, muscle fiber shrinkage, collagen solubilization, connective tissue degradation, and formation of sarcoplasmic and myofibrillar gels collectively determine the final texture and quality of cooked meat [29,30].

Despite the growing interest in both SV and ohmic heating, limited research has investigated the integration of these technologies to improve the physicochemical, textural, and sensory characteristics of camel meat, particularly with the objective of enhancing consumer acceptance. Therefore, the present study aimed to evaluate a novel cooking approach that combines ohmic heating (equipped with a temperature-control system) with SV cooking to improve the physicochemical, textural, and sensory quality of Arabian one-humped camel (*Camelus dromedarius*) meat.

## 2. Materials and methods

### 2.1. Preparation of meat samples

Camel meat samples were obtained from 12 male Saudi Arabian Majaheem camels and purchased from a retail store within 24 h post-slaughter in February 2023. All meat samples were sourced from the flat iron (*infraspinatus*) muscle of the shoulder clod, which is located in the infraspinous fossa of the scapula caudal to the scapular spine and beneath the deltoid muscle, where it forms a major part of the deep shoulder musculature. To ensure consistency, all samples originated from male animals of the same age (6–7 months) and had similar nutritional backgrounds. In the context of Saudi Arabian consumers, the shoulder cut or clod from the camel is considered one of the most preferred and commonly used parts of the animal, alongside the striploin and tenderloin. These specific camel meat cuts are typically utilized for slow roasting preparations or diced and incorporated into wet, braised dish recipes that are popular in the local cuisine. Subsequently, the samples were packed in an isothermal box with ice packs and transported to the Food Process Engineering Laboratory at the College of Food and Agricultural Sciences, King Saud University. Next, the outer fat and connective tissue were removed, and the meat was cut parallel to the direction of the muscle fibers. The meat samples were prepared and shaped to similar sizes (6 cm x 6 cm x 6 cm). The total number of the prepared samples was 120 for both cooking methods; OS vs SV, where 5 samples were allocated randomly for each cooking time (30, 60, 90, and 120 min) per each cooking temperature (70, 80, and 90 ℃) according to Fig 1. The prepared samples were then placed in plastic bags and stored at −20 ℃ till the time of treatment. Prior to cooking, the samples were thawed for 24 hours in a refrigerator at 3 ℃ [31]. The meat sample was placed in a vacuum-sealed cooking pouch (Sous Vide Supreme®, Eades Technology of Appliance, LLC 4347 W., Northwest Hwy Suite 130, Dallas, TX 75220, USA). These pouches are food-safe, high-temperature tolerant, and specially designed to withstand the temperatures involved in SV cooking (Pouch Specifications: Thickness: 85 µm, Heat Resistance: 100 °C for up to 4 hours, Transparency: Semi-transparent, Material Composition: Constructed from layers of food-grade polyethylene and nylon, Safety Certification: Third-party tested and certified free of BPA, phthalates, and lead, and stable at higher temperatures preventing the migration of plastic components into the food.). The pouches were then evacuated using a vacuum sealer (Sous vide Vacuum Sealer VS3000, Eades Technology of Appliance, LLC 4347 W. Northwest Hwy Suite 130, Dallas, TX 75220, USA). This research was conducted following the ethical guidelines endorsed by the Research Ethics Committee (REC) of King Saud University, adhering to all recognized ethical standards for studies involving both animals and human participants. An Institutional Review Board approval was obtained before commencing the research study (IRB No. E-25–10172).

**Fig 1:**
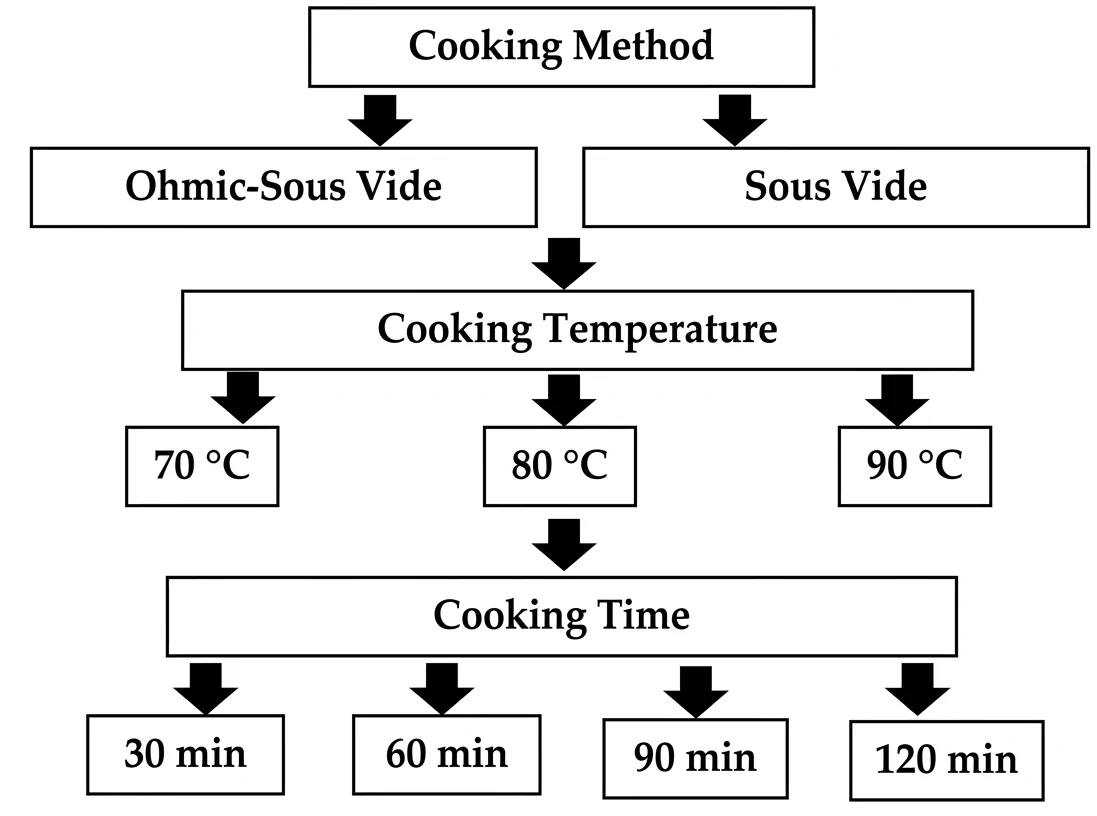
A schematic diagram showing experimental design and treatment combinations.

### 2.2. Cooking methods

This study utilized two distinct cooking methods: OS cooking and traditional SV cooking. The OS cooking method employed an ohmic cooking system developed by Othman, Alfaifi [14]. Fig 2 illustrates the OS cooking system developed at King Saud University. The system consisted of a cuboid Teflon chamber equipped with four cylindrical titanium electrodes. Alternating current (AC; 220 V, 60 Hz) was supplied to generate ohmic heating, while temperature, current, and voltage were continuously monitored using a thermocouple, a current transducer, and a data logger, respectively. Temperature was automatically regulated using a proportional, integral, and derivative (PID) controller to ensure precise control throughout the cooking process. The camel meat samples were placed in the OS cooking system for this process. For the SV cooking method, the samples were immersed in a water bath (GFL Water Bath, Model 1083, GFL Technology Laboratory Equipment-LAUDA, Laudaplatz 1–97922 Lauda Königshofen, Germany) and subjected to various combinations of temperatures (70, 80, and 90 °C) and durations (30, 60, 90, and 120 min), paralleling the conditions used in the OS method.

**Fig 2:**
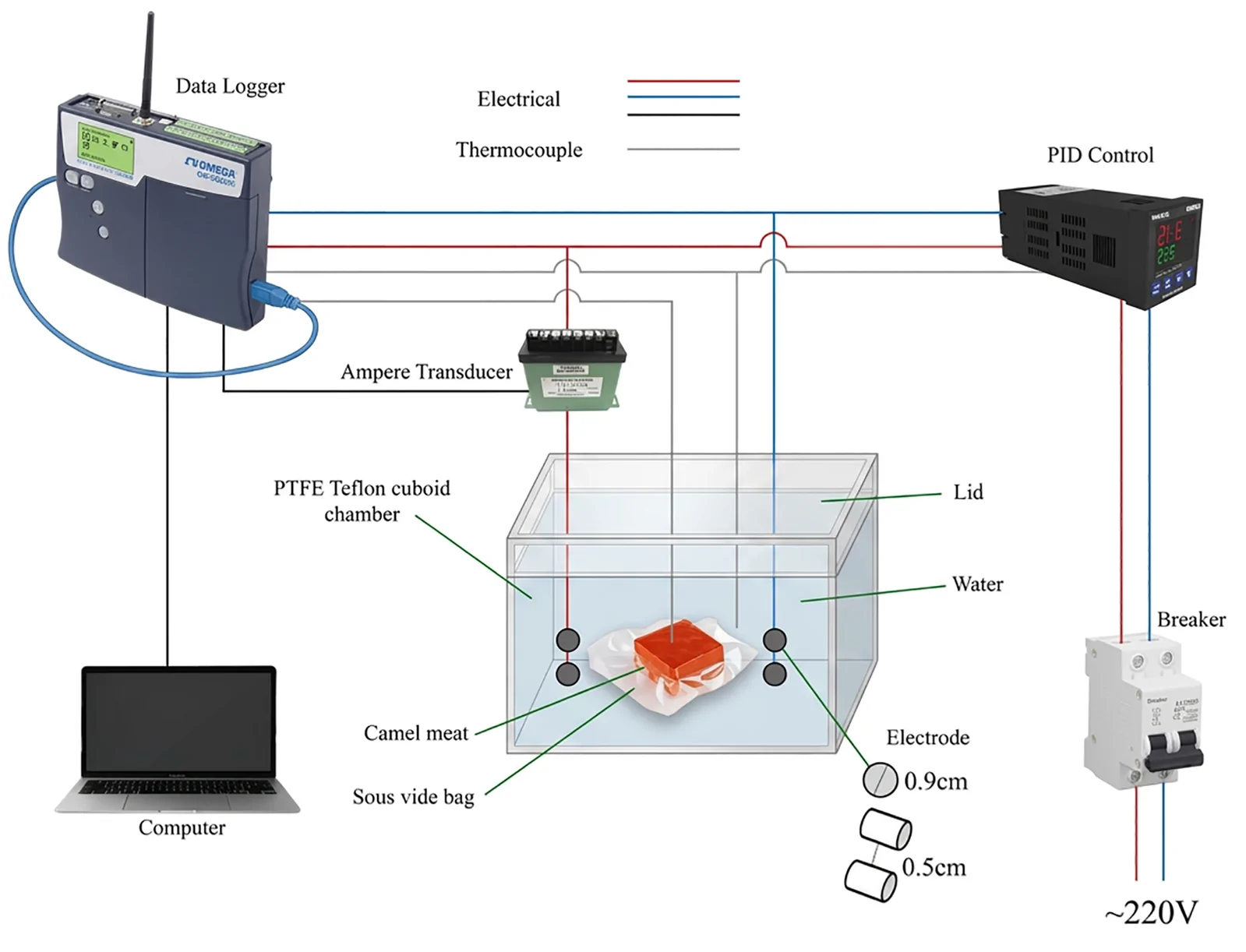
The developed ohmic-sous vide cooking system Othman, Alfaifi [14].

### 2.3. Quality characteristics measurements

#### 2.3.1. Color.

Fresh and cooked meat color was measured using a colorimeter (CR400 Chroma Meter, Konica Minolta, Inc., Marunouchi, Chiyoda, Tokyo, Japan: Measuring aperture: 8 mm; Illuminant: CIE D65; Observer angle: CIE 2° Standard Observer), at room temperature (22 ± 2 °C). The measurements for the camel meat sample were conducted after a period of 30 min of blooming, following which the samples were placed in the optical port of the device, and the measurements were obtained. Basic color components include lightness (L*), redness (a*), and yellowness (b*). Some derived properties of the color were also obtained, such as the color change, color hue angle, and chroma [32]. The means and standard error were calculated from five replicates.

#### 2.3.2. pH.

The measurement of *pH* level of the camel meat samples was performed before and after cooking using a pH-Meter connected to a specialized FC2323 meat pH electrode with a stainless-steel piercing blade (Professional Foodcare Portable Meat pH Meter - HI98163, Hanna Instrument, 18 Fiveways Blvd., Keysborough, VIC 3173, Australia). The sensor connected to the device was used to permeate the meat sample, and readings were recorded [33]. Three readings from five replicates were obtained to calculate the means and standard error values.

#### 2.3.3. Water activity.

Water activity (a_w_) is defined as the ratio of the vapor pressure of water in the sample to that of pure water at the same temperature and pressure. A representative 20 g portion of cooked camel meat was homogenized, and five replicates were analyzed to calculate the mean and standard error [6]. Water activity was measured using a water activity meter (AquaLab Series 3, Decagon Devices Inc., Pullman, WA, USA), which was calibrated before each measurement using standard salt solutions of known water activity. The homogenized sample was placed in the sample chamber and allowed to equilibrate with the chamber atmosphere until a stable reading was obtained [6]. All measurements were performed at a controlled temperature of 25°C, as water activity is temperature dependent.

#### 2.3.4. Cooking loss.

The cooking loss (CL) was determined as the change in weight of camel meat samples before and after cooking. The weight differences between samples were measured using a precision balance (0.01 g sensitivity; model 2100/C/2, Radwag Wagi Elektroniczne, Toruńska 5, Radom 26–600, Poland).


$$\begin{array}{c} \text{Cooking loss }(\%)=\frac{\text{Fresh camel meat }(\text{g})-\text{Cooked camel meat}(\text{g})}{\text{Fresh camel meat }(\text{g})}\times \text{100} \end{array}$$

#### 2.3.5. Shear force.

The shear force of the cooked camel meat samples was measured using a texture analyzer (TA-HDi, Model HD3128, Stable Micro Systems Ltd., Surrey, UK) equipped with Texture Expert Exceed software, version 2.05. Before analysis, the instrument was calibrated according to the manufacturer’s instructions using the appropriate load cell and calibration weight. The cooked samples previously used for cooking-loss determination were cooled to room temperature 22 ± 2 °C [34]. Five cylindrical cores, each 1.27 cm in diameter, were removed from each sample parallel to the longitudinal orientation of the muscle fibers using a manual coring device. Each core was sheared perpendicular to the muscle-fiber direction using a Warner-Bratzler V-shaped blade at a crosshead speed of 1.5 mm/s. The blade traveled a sufficient distance to completely shear each core. The maximum force required to shear each core was recorded in newtons (N), and the mean value obtained from the five cores was considered the shear-force value for each meat sample [35,36].

### 2.4. Sensory attributes

The sensory evaluation was conducted in a sensory analysis laboratory at the College of Food and Agricultural Sciences, King Saud University, under controlled room conditions (22 ± 2 °C). Thirty untrained consumers, comprising university staff, postgraduate students, and researchers who were regular consumers of meat products, participated in the evaluation. Prior to participation, all panelists provided written informed consent. The evaluation was conducted in three consecutive sessions of ten panelists each, with each session lasting approximately 30 min. Camel meat samples were cooked according to the designated temperature–time combinations, cut into approximately 1.5 cm³ cubes, and identified using randomized three-digit codes. The samples were served warm (internal temperature of approximately 70 °C), wrapped in aluminum foil, and presented in disposable plastic containers with lids on numbered serving plates. Panelists evaluated the samples independently for overall acceptability, tenderness, flavor, and juiciness. They were instructed to refrain from eating or smoking for at least 2 h before the evaluation. Drinking water was provided for palate cleansing between samples, and communication among panelists during the evaluation was not permitted.

Consumer acceptance was assessed using an 8-point hedonic scale adapted from the American Meat Science Association [37], where 1 = dislike extremely, 2 = dislike very much, 3 = dislike moderately, 4 = dislike slightly, 5 = like slightly, 6 = like moderately, 7 = like very much, and 8 = like extremely. Unlike the conventional 9-point hedonic scale, the 8-point scale intentionally omits a neutral (“neither like nor dislike”) category to encourage panelists to express either a positive or negative preference and to minimize central tendency bias, thereby improving discrimination among treatments [38–41].

### 2.5. Statistical analysis

The experiment was conducted using a completely randomized design (CRD) with a three-factor factorial arrangement (2 × 3 × 4). The three fixed factors were cooking method (OS and traditional SV), cooking temperature (70, 80, and 90°C), and cooking time (30, 60, 90, and 120 min). The effects of the three main factors and their two-way interactions (cooking method × temperature, cooking method × time, and temperature × time), as well as their three-way interaction (cooking method × temperature × time), were evaluated.

Prior to statistical analysis, data were examined for normality using the Shapiro–Wilk test. All response variables satisfied the assumption of normality. Data were analyzed using the General Linear Model (GLM) procedure of IBM SPSS Statistics for Windows, Version 27.0 (IBM Corp., Armonk, NY, USA), with cooking method, cooking temperature, and cooking time specified as fixed effects. When significant treatment effects were detected (P ≤ 0.05), differences among means were separated using the least significant difference (LSD) multiple comparison test. Results are presented as means ± standard error of the mean (SEM) unless otherwise stated, and statistical significance was declared at P ≤ 0.05.

## 3. Results

### 3.1. The descriptive statistics

Table 1 summarizes the descriptive statistics, including the mean, minimum and maximum values, range, median, and coefficient of variation (CV), for the physical and textural characteristics of fresh camel meat. These parameters comprise the instrumental color coordinates including lightness (L*), redness (a*), and yellowness (b*) as well as pH, water activity (a_w_), and shear force (SF). The inclusion of the CV provides a standardized measure of relative variability, enabling comparison of data dispersion among variables with different units and scales. Lower CV values indicate greater uniformity and consistency among samples, whereas higher CV values reflect increased variability, thereby providing additional insight into the homogeneity and reliability of the measured quality attributes.

**Table 1:** The descriptive statistics of color and physicochemical properties of fresh camel meat (Mean ± SD) (*N = 12*).

Property	Mean	Lower value	Upper value	Range	Median	CV
Lightness (L*)	50.98 ± 0.43	49.92	53.20	3.28	50.72	2.26
Redness (a*)	17.98 ± 0.35	17.50	18.32	0.82	18.08	1.98
Yellowness (b*)	4.65 ± 0.20	4.56	4.87	0.31	4.62	4.00
*pH*	5.92 ± 0.03	5.87	5.94	0.07	5.92	0.47
Water activity (a_w_)	0.99 ± 0.02	0.99	0.99	0.00	0.99	0.08
Shear force (N)	70.20 ± 0.47	69.15	71.29	2.14	70.18	0.97

* CV, coefficient of variation; *N,* total number of samples.

The mean redness (a*) value was 17.98, with values ranging from 17.50 to 18.32 and a median of 18.08, indicating a relatively uniform distribution of redness among the samples. Yellowness (b*) exhibited a mean value of 4.65, with minimum and maximum values of 4.56 and 4.87, respectively. Although the absolute range of b* was relatively small, it showed the highest coefficient of variation among the color coordinates, indicating that yellowness was the most variable color attribute relative to its mean. In contrast, the lower CV values observed for L* and a* suggest greater consistency in lightness and redness across the fresh camel meat samples.

The mean pH was 5.92, with values confined to a narrow interval (5.87–5.94), corresponding to a range of only 0.07 pH units. This limited variation, reflected by a very low CV, demonstrates the high uniformity of muscle acidity among the sampled meat and indicates consistent post-mortem biochemical changes. Similarly, water activity (a_w_) was highly stable, with both the mean and median equal to 0.99 and an extremely low coefficient of variation, confirming the uniform moisture availability across the samples.

Shear force, an indicator of meat tenderness, had a mean value of 70.20 N, with values ranging from 69.15 to 71.29 N and a median of 70.18 N. The close agreement between the mean and median, together with the low CV, indicates a symmetrical distribution and minimal variability in instrumental tenderness. Additionally, the consistently low CV values observed for most measured traits demonstrate the high degree of sample homogeneity and experimental consistency, thereby strengthening the reliability of the dataset and providing a robust basis for subsequent correlation and multivariate analyses.

### 3.2. The quality characteristics of camel meat

#### 3.2.1. The color components of camel meat.

Table 2 shows the effects of the cooking method (OS and SV), cooking temperature (70, 80, 90 °C), and cooking time (30, 60, 90, 120 min) on the color components (lightness, redness, yellowness, color change, hue angle, and chroma) of camel meat. The three-way interaction among cooking method, cooking temperature, and cooking time significantly affected most color attributes, including lightness (L*; p < 0.001), yellowness (b*; p = 0.01), total color change (ΔE; p < 0.001), and chroma (C*; p < 0.001). In contrast, redness (a*) and hue angle (h°) were not significantly influenced (p > 0.05). The lightness (L*) showed values ranging from 54.73 to 30.28, redness (a*) ranging from 13.67 to 5.65, and yellowness (b*) ranging from 9.19 to 20.31. These findings provided valuable insights into how different cooking techniques and parameters impact the visual appearance and color perception of camel meat. S1 File (Figures A-C) demonstrates the influence of cooking method on color components; lightness (L*), redness (a*), and yellowness (b*) of camel meat at different cooking temperatures and times. The results showed that meat cooked using the SV method had higher (p < 0.001) lightness values compared to the OS method. Specifically, when cooked at a temperature of 90 °C and a duration of 120 min. A significant three-way interaction among cooking method, cooking temperature, and cooking time (CMD × CTM × CTI, p < 0.001) was observed for lightness (L*), with SV-cooked samples exhibiting approximately 2.04% higher lightness than OS-cooked samples. It was also observed that increasing cooking temperature (CTM, p < 0.001) and cooking time (CTI, p < 0.001) significantly reduced the lightness (L*) of camel meat. On the other hand, the redness color component was observed to be higher (p < 0.001) in meat cooked using the SV method compared to the OS system. Camel meat cooked by the SV method tended to exhibit higher redness (a*) than that cooked by the OS method, reaching an approximately 6.46% difference at 90 °C for 120 min; however, the three-way interaction among cooking method, temperature, and time was not significant (CMD × CTM × CTI, p > 0.05). Furthermore, increasing cooking temperature (CTM, p < 0.001) and cooking time (CTI, p < 0.001) significantly reduced the redness (a*) of camel meat across both cooking methods.

**Table 2:** Effect of cooking method (CMD), cooking temperature (CTM), and cooking time (CTI) on color components of camel meat (N = 120).

Treatment	*n*	Color Components					
		L*	a*	b*	Δ𝐄	${𝐡}_{𝐚𝐛}^{*}$	${𝐂}_{𝐚𝐛}^{*}$
**Cooking Method:**							
Sous vide cooking	60	39.70	9.33	13.10	16.05	54.37	16.34
Ohmic-sous vide cooking	60	39.12	8.11	17.46	18.39	64.73	19.39
*SEM*		0.08	0.06	0.06	0.07	0.19	0.06
*p-value*		< 0.001	< 0.001	< 0.001	< 0.001	< 0.001	< 0.001
**Cooking temperature (°C):**							
70	20	43.73^a^	10.35^a^	13.93^c^	14.56^c^	52.85^c^	17.60^b^
80	20	38.74^b^	8.52^b^	15.10^b^	17.04^b^	59.87^b^	17.53^b^
90	20	35.77^c^	7.27^c^	16.81^a^	20.02^a^	65.94^a^	18.45^a^
*SEM*		0.10	0.07	0.07	0.09	0.24	0.07
*p-value*		< 0.001	< 0.001	< 0.001	< 0.001	< 0.001	< 0.001
**Cooking time (min):**							
30	5	46.26^a^	10.64^a^	13.18^d^	12.82^d^	50.30^d^	17.23^d^
60	5	41.72^b^	9.16^b^	14.74^c^	15.07^c^	57.59^c^	17.52^c^
90	5	36.64^c^	7.95^c^	15.99^b^	18.78^b^	63.11^b^	17.98^b^
120	5	33.02^d^	7.11^d^	17.22^a^	22.15^a^	67.21^a^	18.72^a^
*SEM*		0.12	0.08	0.08	0.10	0.28	0.08
*p-value*		< 0.001	< 0.001	< 0.001	< 0.001	< 0.001	< 0.001
**Interactions:**		***p-value***					
CMD x CTM		< 0.001	< 0.001	< 0.001	< 0.001	< 0.001	< 0.001
CMD x CTI		< 0.001	< 0.001	< 0.001	< 0.001	< 0.001	NS
CTM x CTI		< 0.001	NS	< 0.001	< 0.001	< 0.001	NS
CMD x CTM x CTI		< 0.001	NS	0.01	< 0.001	NS	< 0.001

a,b,c,dMeans within the same column with different superscripts are significantly different. ΔE; Color change, ${\text{h}}_{\text{ab}}^{*}$, Hue angle, ${\text{C}}_{\text{ab}}^{*}$; Chroma; *n*, number of samples; SEM, standard error of mean; P-value, probability level (significant when *P ≤ 0.05*); NS, not significantly different.

In contrast to the other color attributes, camel meat cooked using the OS method exhibited significantly higher yellowness (b*) than that cooked using the SV method (CMD, p < 0.001). The greatest difference was observed at 90 °C for 120 min, where the b* value of OS-cooked meat was approximately 20.05% higher than that of SV-cooked meat, as reflected by the significant three-way interaction among cooking method, temperature, and time (CMD × CTM × CTI, p = 0.01). Furthermore, yellowness (b*) increased significantly with increasing cooking temperature (CTM, p < 0.001) and holding time (CTI, p < 0.001), regardless of the cooking method.

Increasing cooking temperature significantly influenced all color attributes, including lightness (L; p < 0.001), redness (a; p < 0.001), yellowness (b*; p < 0.001), total color change (ΔE; p < 0.001), hue angle (h°; p < 0.001), and chroma (C*; p < 0.001). Specifically, lightness and redness decreased, whereas yellowness, total color change, hue angle, and chroma increased as cooking temperature increased. Increasing cooking time significantly influenced all color attributes (p < 0.001). Specifically, yellowness (b*), total color change (ΔE), hue angle (h°), and chroma (C*) increased, whereas lightness (L*) and redness (a*) decreased as cooking time increased.

Compared with the OS method, SV cooking produced camel meat with significantly higher lightness (L*; p < 0.001) and redness (a*; p < 0.001) but lower yellowness (b*; p < 0.001), resulting in a lighter and redder overall color appearance.

The significant interaction between cooking method and cooking temperature (CMD × CTM) indicates that the effect of cooking temperature on camel meat color depended on the cooking method employed. This interaction significantly affected all color attributes, including lightness (L*; p < 0.001), redness (a*; p < 0.001), yellowness (b*; p < 0.001), total color change (ΔE; p < 0.001), hue angle (h°; p < 0.001), and chroma (C*; p < 0.001).

The interaction between cooking method and cooking time (CMD × CTI) significantly affected all color attributes except chroma, including lightness (L*; p < 0.001), redness (a*; p < 0.001), yellowness (b*; p < 0.001), total color change (ΔE; p < 0.001), and hue angle (h°; p < 0.001), whereas chroma (C*) was not significantly affected (p > 0.05). Similarly, the interaction between cooking temperature and cooking time (CTM × CTI) significantly influenced lightness (L*; p < 0.001), yellowness (b*; p < 0.001), total color change (ΔE; p < 0.001), and hue angle (h°; p < 0.001), but had no significant effect on redness (a*) or chroma (C*) (p > 0.05).

The significant two-way interactions (CMD × CTM, CMD × CTI, and CTM × CTI), together with the significant three-way interaction (CMD × CTM × CTI), indicate that camel meat color development was influenced by the combined effects of cooking method, temperature, and time. The three-way interaction significantly affected lightness (L*), yellowness (b*), total color change (ΔE), and chroma (C*), whereas redness (a*) and hue angle (h°) were not significantly influenced.

Fig 3 illustrates the color characteristics of camel meat cooked by SV and OS under different combinations of cooking temperatures (70, 80, and 90 °C) and cooking times (30, 60, 90, and 120 min). Both cooking methods produced a progressive transition in meat color as cooking temperature and time increased. Samples cooked at 70 °C for 30 min retained a relatively light reddish-brown appearance, indicating less extensive pigment denaturation. As cooking temperature increased from 70 to 90 °C and cooking time increased from 30 to 120 min, the meat gradually became darker and browner, reflecting the progressive thermal denaturation of myoglobin and the formation of brown pigments. At 70 °C, the color changes between OS and SV were relatively small. Both methods produced meat with a pinkish- to light-brown appearance, although the OS samples generally appeared slightly more uniform in color than the corresponding SV samples. While at 80 °C, browning became more pronounced in both cooking methods. The OS samples tended to exhibit a more homogeneous brown color over the entire surface, whereas several SV samples displayed greater color heterogeneity, with lighter and darker regions remaining visible. This suggests that OS provided more uniform heat distribution during cooking. At 90 °C, particularly after 90 and 120 min, all samples developed the characteristic brown color associated with well-cooked meat. However, the OS-treated samples generally appeared to have a deeper and more uniform brown coloration than the SV-treated samples, while SV samples retained slight variations in surface color and localized lighter areas. These visual differences are consistent with the more rapid and uniform volumetric heating generated during ohmic processing. Cooking time also had a clear influence on appearance. Within each cooking temperature, extending the cooking duration resulted in progressively darker surfaces and a reduction in reddish hue. The greatest visual changes occurred between 30 and 90 min, whereas the difference between 90 and 120 min was comparatively smaller, suggesting that most pigment transformation had already occurred by the longer cooking periods.

**Fig 3:**
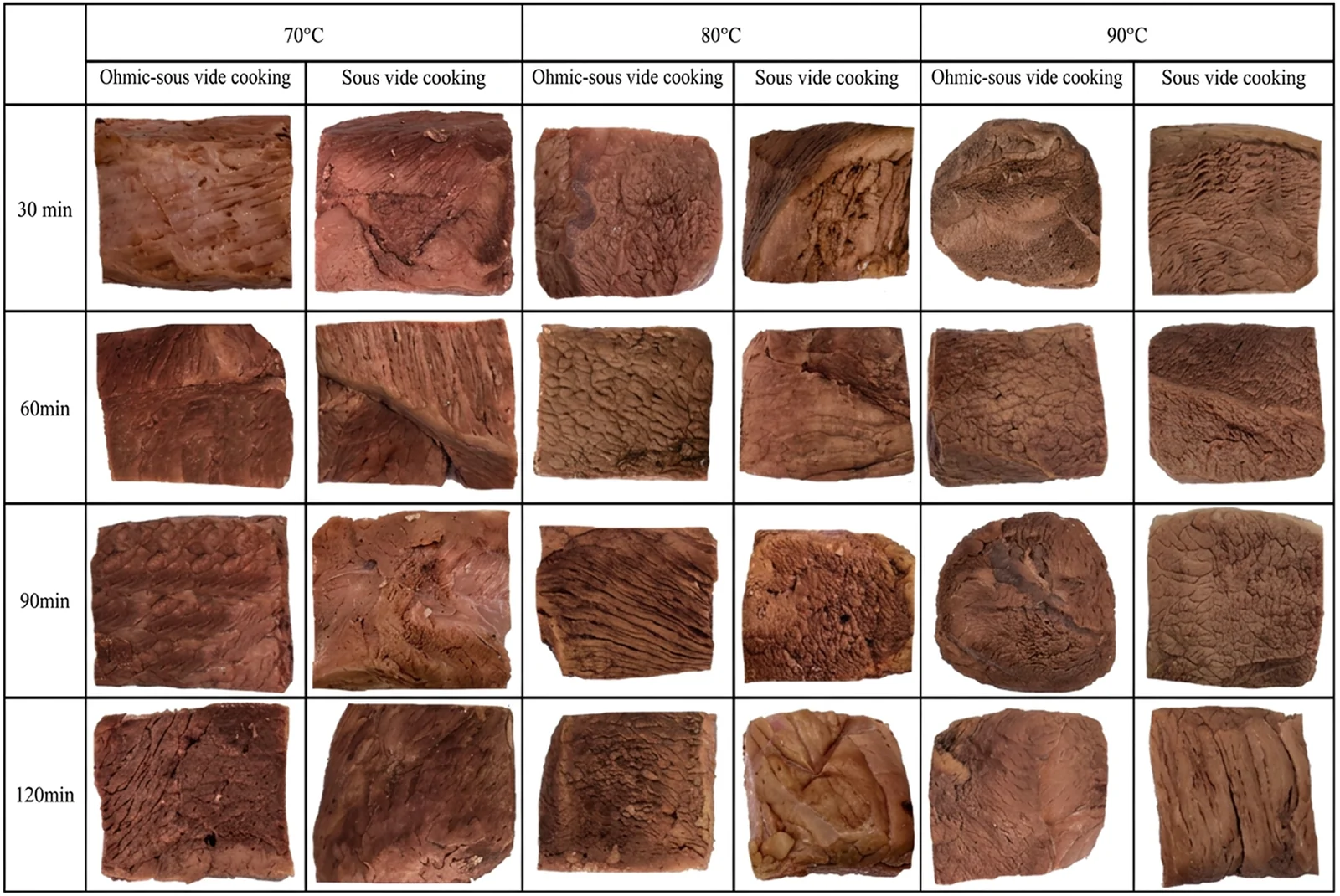
Color of cooked camel meat using sous vide and ohmic-sous vide cooking methods at different temperatures and times.

#### 3.2.2. The physicochemical properties of camel meat.

The effects of cooking method (CMD), cooking temperature (CTM), and cooking time (CTI) on the physicochemical properties of camel meat are presented in Table 3. Cooking method, cooking temperature, and cooking time significantly (p < 0.001) affected all evaluated parameters, including pH, cooking loss (CL), water activity (a_w_), and shear force (SF).

**Table 3:** Effect of cooking method (CMD), cooking temperature (CTM), and cooking time (CTI) on the physicochemical properties of camel meat (N = 120).

Treatment	*n*	Physicochemical Characteristics			
		pH	CL (%)	a_w_	SF (N)
**Cooking Method:**					
Sous vide cooking	60	6.11	34.25	0.981	29.87
Ohmic-sous vide cooking	60	6.16	40.10	0.983	24.31
*SEM*		0.004	0.14	< .001	0.13
*p-value*		< 0.001	< 0.001	< 0.001	< 0.001
**Cooking temperature (°C):**					
70	20	6.06^c^	31.96^c^	0.99^a^	34.84^a^
80	20	6.13^b^	37.19^b^	0.98^b^	26.85^b^
90	20	6.22^a^	42.36^a^	0.97^c^	19.58^c^
*SEM*		0.005	0.17	< .001	0.16
*p-value*		< 0.001	< 0.001	< 0.001	< 0.001
**Cooking time (min):**					
30	5	6.00^d^	29.62^d^	0.987^a^	37.27^a^
60	5	6.11^c^	36.19^c^	0.984^b^	29.49^b^
90	5	6.17^b^	39.61^b^	0.980^c^	24.01^c^
120	5	6.26^a^	43.28^a^	0.977^d^	17.59^d^
*SEM*		0.006	0.19	< .001	0.18
*p-value*		< 0.001	< 0.001	< 0.001	< 0.001
**Interactions:**				***p-value***	
CMD x CTM		0.002	< 0.001	< 0.001	0.03
CMD x CTI		NS	0.008	< 0.001	< 0.001
CTM x CTI		0.01	< 0.001	< 0.001	< 0.001
CMD x CTM x CTI		0.03	< 0.001	NS	< 0.001

a,b,c,d Means within the same column with different superscripts are significantly different. CL: cooking loss, *n*, number of samples; SEM, standard error of mean; P-value, probability level (significant when *P ≤ 0.05*); NS, not significantly different.

Cooking method significantly influenced pH, cooking loss, water activity, and shear force (p < 0.001 for all parameters). Camel meat cooked using the OS method exhibited a significantly higher pH (6.16) than meat cooked using conventional SV (6.11). Cooking loss was also significantly greater in OS-treated samples (40.10%) than in SV-cooked samples (34.25%). Likewise, water activity differed significantly between the two cooking methods, with mean values of 0.983 and 0.981 for OS and SV, respectively. In contrast, shear force was significantly lower in OS-cooked samples (24.31 N) than in SV-cooked samples (29.87 N).

Cooking temperature had a significant effect on all measured physicochemical properties (p < 0.001). The pH of camel meat increased progressively with increasing cooking temperature, from 6.06 at 70°C to 6.22 at 90°C. All temperature treatments differed significantly from one another (p < 0.05). Cooking loss also increased significantly with increasing temperature, reaching its lowest value at 70°C and highest value at 90°C. Similarly, shear force decreased significantly as cooking temperature increased, whereas water activity declined progressively from 70 to 90°C, with significant differences observed among all temperature treatments (p < 0.05).

Cooking temperature significantly (p < 0.001) affected pH, cooking loss (CL), water activity (a_w_), and shear force (SF). The pH increased progressively with increasing cooking temperature, from 6.06 at 70°C to 6.22 at 90°C, with all temperature treatments differing significantly (p < 0.05) from one another. Cooking loss also increased significantly with temperature, ranging from 31.96% at 70°C to 42.36% at 90°C. In contrast, water activity decreased significantly from 0.99 at 70°C to 0.97 at 90°C. Likewise, shear force declined significantly with increasing cooking temperature, with the highest value recorded at 70°C (34.84 N) and the lowest at 90°C (19.58 N).

Cooking time significantly influenced pH, cooking loss, water activity, and shear force (p < 0.001). Increasing the cooking time from 30 to 120 min resulted in a progressive increase in pH from 6.00 to 6.26. Cooking loss increased significantly with prolonged cooking duration, with the lowest value observed after 30 min (29.62%) and the highest after 120 min (43.28%). All cooking-time treatments differed significantly (p < 0.05) from one another. Water activity decreased significantly from 0.987 at 30 min to 0.977 at 120 min, whereas shear force decreased from 37.27 N at 30 min to 17.59 N at 120 min.

Significant interaction effects were also observed among the experimental factors. The interaction between cooking method and temperature (CMD × CTM) significantly affected pH (p = 0.002), cooking loss (p < 0.001), water activity (p < 0.001), and shear force (p = 0.03). The interaction between cooking method and time (CMD × CTI) significantly influenced cooking loss (p = 0.008), water activity (p < 0.001), and shear force (p < 0.001), whereas its effect on pH was not significant (p > 0.05). The interaction between cooking temperature and time (CTM × CTI) significantly affected all measured physicochemical properties (p ≤ 0.01). Furthermore, the three-way interaction among cooking method, cooking temperature, and cooking time (CMD × CTM × CTI) significantly influenced pH (p = 0.03), cooking loss (p < 0.001), and shear force (p < 0.001), whereas no significant effect was observed for water activity (p > 0.05).

The impact of cooking methods at different combinations of cooking temperatures and durations on quality characteristics of camel meat, including pH, cooking loss, water activity, and shear force are presented in the S1 File (Figures D-G). The pH values spanned a range of 5.87 to 6.37, indicating variations in the acidity levels of the meat, which were influenced by the cooking method and conditions. The pH of camel meat increased significantly with the cooking method (CMD, p < 0.001), increasing cooking temperature (CTM, p < 0.001), and increasing cooking time (CTI, p < 0.001). Additionally, when comparing the pH of meat cooked using OS cooking and SV cooking, it was observed that OS cooking resulted in a higher (p < 0.001) pH than SV cooking, specifically at a temperature of 90 °C and a cooking time of 120 min. The pH value for OS cooking was measured as 6.37, while for SV cooking was 6.31.

The cooking loss exhibited a range of 21.93% to 52.7%, signifying discrepancies in moisture retention and loss during the cooking process. Cooking loss increased significantly with increasing cooking temperature (CTM, p < 0.001) and cooking time (CTI, p < 0.001) under both the SV and OS cooking methods, with a significant effect of cooking method also observed (CMD, p < 0.001). Furthermore, it was found that camel meat cooked using OS cooking experienced a higher (p < 0.001) cooking loss compared to SV cooking. Specifically, at a temperature of 90 °C and a cooking time of 120 min.

The water activity of cooked camel meat using the SV and OS methods decreased from 0.995 and 0.993 at 70 ºC to 0.981 and 0.973, respectively. The water activity of cooked camel meat decreased significantly as cooking temperature (CTM, p < 0.001) and cooking time (CTI, p < 0.001) increased under both the SV and OS cooking methods, with a significant main effect of cooking method also observed (CMD, p < 0.001). Increasing cooking temperature and cooking time significantly reduced the shear force of cooked camel meat under both cooking methods (CTM, p < 0.001; CTI, p < 0.001), with a significant effect of cooking method also observed (CMD, p < 0.001).

The interactions between cooking method and cooking temperature (CMD × CTM) and between cooking temperature and cooking time (CTM × CTI) significantly influenced all measured physicochemical characteristics. Specifically, the CMD × CTM interaction significantly affected pH (p = 0.002), cooking loss (p < 0.001), water activity (p < 0.001), and shear force (p = 0.03), whereas the CTM × CTI interaction significantly influenced pH (p = 0.01), cooking loss (p < 0.001), water activity (p < 0.001), and shear force (p < 0.001). The significant interaction between cooking method and cooking time (CMD × CTI) indicates that the effects of cooking time on cooking loss (p = 0.008), water activity (p < 0.001), and shear force (p < 0.001) depended on the cooking method employed, while no significant interaction was observed for pH (p > 0.05). Notably, water activity was the only parameter that did not exhibit a significant three-way interaction CMD × CTM × CTI (p > 0.05), indicating that the combined influence of cooking method, cooking temperature, and cooking time did not result in a significant effect on water activity.

#### 3.2.3. The sensory evaluation of camel meat.

Table 4 shows the effect of cooking method on the sensory characteristics (overall acceptability, tenderness, flavor, and juiciness) of camel meat at different combinations of temperatures (70 °C, 80 °C, and 90 °C) and cooking times (30, 60, 90, and 120 minutes). Significant differences between the SV and OS cooking methods were observed for overall acceptability (p < 0.001) and tenderness (p = 0.01), whereas flavor and juiciness were not significantly affected (p > 0.05). Ohmic-sous vide cooking received higher scores in overall acceptability (5.34), tenderness (5.10), flavor (5.05), and juiciness (4.50) compared to SV cooking, which had ratings of 4.95, 4.88, 5.09, and 4.50, respectively. The present findings demonstrate that cooking temperature significantly affected all evaluated sensory characteristics, including overall acceptability, tenderness, flavor, and juiciness (all p < 0.001). Increasing cooking temperature enhanced tenderness and flavor but reduced juiciness, reflecting the combined effects of heat-induced connective tissue solubilization and increased moisture loss during cooking. Consequently, these outcomes were favorable for both cooking methods. Similarly, increasing cooking time significantly enhanced overall acceptability, tenderness, and flavor, while reducing juiciness (all p < 0.001). The improved tenderness and flavor associated with prolonged cooking likely outweighed the reduction in juiciness, contributing to the higher overall acceptance scores. The flavor of cooked camel meat improved significantly with increasing cooking temperature (CTM, p < 0.001) and cooking time (CTI, p < 0.001), likely due to enhanced Maillard reactions and lipid-derived flavor development during prolonged thermal processing. The juiciness of cooked camel meat decreased significantly as cooking temperature (CTM, p < 0.001) and cooking time (CTI, p < 0.001) increased, likely due to greater moisture loss associated with prolonged exposure to higher temperatures, which holds true for both cooking methods. Specifically, when camel meat was cooked using SV at 90 °C for 120 minutes, the juiciness score of camel meat cooked by OS surpassed that cooked by SV by almost 48.89%. Both the SV and OS methods achieved the highest acceptance score of 7.9 when using a cooking temperature of 90 °C and a cooking time of 120 minutes. Cooking temperature and time play crucial roles in influencing changes in the structural and quality attributes of camel meat.

**Table 4:** Effect of cooking method (CMD), cooking temperature (CTM) and cooking time (CTI) on sensory characteristics of camel meat (*N = 120*).

Treatment	*n*	Sensory Characteristics			
		Overall acceptability	Tenderness	Flavor	Juiciness
**Cooking Method:**					
Sous vide cooking	60	4.95	4.88	5.09	4.50
Ohmic-sous vide cooking	60	5.34	5.10	5.05	4.70
*SEM*		0.05	0.05	0.05	0.05
*p-value*		< 0.001	0.01	NS	NS
**Cooking temperature (°C):**					
70	20	4.04^c^	3.83^c^	4.06^c^	5.54^a^
80	20	5.16^b^	5.13^b^	5.01^b^	4.56^b^
90	20	6.23^a^	5.99^a^	6.15^a^	3.81^c^
*SEM*		0.06	0.07	0.06	0.06
*p-value*		< 0.001	< 0.001	< 0.001	< 0.001
**Cooking time (min):**					
30	5	3.56^d^	2.93^d^	3.46^d^	6.10^a^
60	5	4.55^c^	4.62^c^	4.65^c^	5.03^b^
90	5	5.73^b^	5.75^b^	5.60^b^	4.20^c^
120	5	6.72^a^	6.62^a^	6.58^a^	3.22^d^
*SEM*		0.07	0.08	0.07	0.07
*p-value*		< 0.001	< 0.001	< 0.001	< 0.001
**Interactions:**	***p-value***				
CMD x CTM		NS	NS	NS	NS
CMD x CTI		NS	NS	NS	NS
CTM x CTI		NS	< 0.001	0.01	NS
CMD x CTM x CTI		NS	NS	NS	NS

a,b,c,d Means within the same column with different superscripts are significantly different, *n*, number of samples; SEM, standard error of mean; P-value, probability level (significant when *P ≤ 0.05*); NS, not significantly different.

Increasing cooking temperature and cooking time significantly improved the overall acceptability of cooked camel meat (CTM, p < 0.001; CTI, p < 0.001). The interactions between cooking method and cooking temperature (CMD × CTM), cooking method and cooking time (CMD × CTI), and the three-way interaction (CMD × CTM × CTI) had no significant effects on any of the evaluated sensory attributes (p > 0.05). In contrast, the interaction between cooking temperature and cooking time (CTM × CTI) significantly affected tenderness (p < 0.001) and flavor (p = 0.01), whereas overall acceptability and juiciness were not significantly influenced (p > 0.05).

## 4. Discussion

### 4.1. The descriptive statistics

The descriptive statistics (mean, minimum, maximum, range, median, and coefficient of variation) for the physical and textural properties of fresh camel meat, including color parameters; lightness (L*), redness (a*), and yellowness (b*), pH, water activity (a_w_), and shear force (SF), were generally comparable with those reported in previous studies [6,42] that examined the influence of cooking methods on the physical and sensory properties, and the chemical composition and structural characteristics of the Arabian camel (*Camelus dromedarius*) meat, respectively. The pH of the fresh camel meat was 5.92, which agrees with previous research studies [6,42,43]. According to Kadim, Mahgoub [44], the pH value of Arabian camel meat is between 5.5 and 6.6 and any variation in its value could be attributed to differences in glycogen content of meat. It’s known that the main drive of post-mortem pH changes in meat is the biochemical process of glycolysis, modulated by factors like temperature, ante-mortem stress, species, and muscle type.

Water activity of the camel meat reported in this study was similar to that of Hobani, Othman [6]. Additionally, the shear force value was also in agreement with other reported data of studies [45,46]. Generally, a lower coefficient of variation (CV) indicates lower relative variability and is often considered favorable as it appeared here. It suggests that the data points are relatively close to the mean and have less dispersion or spread. These smaller CV values indicate more consistency and less variability within the dataset.

### 4.2. The quality characteristics of camel meat

#### 4.2.1. The color components of camel meat.

The collective indication of color components in meat refers to the overall color characteristics that can be observed and measured. Assessing and analyzing the overall color attributes of meat samples will provide insights into their visual appearance and potential quality characteristics.

The treatments (cooking method, cooking temperature, and cooking time) significantly affected most color attributes, including lightness (L*), yellowness (b*), total color change (ΔE), and chroma (C*), whereas redness (a*) and hue angle (h°) were not significantly influenced by the treatments, indicating that these color parameters were less sensitive to the combined effects of the processing conditions.

It was observed that increasing cooking temperature (CTM) and cooking time (CTI) significantly reduced the lightness (L*) of camel meat which can be attributed to the denaturation of fiber proteins, that leads to increased fat oxidation and myoglobin degradation [10,47,48]. The resulting color of cooked camel meat was a light brown, which aligns with other findings [48].

Camel meat cooked by the SV method tended to exhibit higher redness (a*) than that cooked by the OS method at 90 °C for 120 min; however, the three-way interaction (CMD × CTM × CTI) was not significant which suggests that the meat processed using OS cooking exhibits a broader range of redness compared to the meat processed using SV cooking. The disparity in redness can be attributed to the use of an electric current in OS cooking, which passes through the cooking medium, whereas SV cooking involves heat energy transfer via convection. Moreover, during OS cooking, the generated electric field induces the movement of ions within the surrounding water medium and potentially within the meat itself through small leakage currents and capacitive effects across the pouch, as opposed to SV cooking [49]. These findings are consistent with previous reported studies [10,47].

The redness (a*) of camel meat across both cooking methods is significantly reduced as a result of increasing CTM and CTI. This is primarily attributed to the chemical reactions involving the myoglobin pigment. These outcomes are comparable with previous research studies [48,50].

Unlike lightness and redness, yellowness (b*) was more strongly influenced by the cooking method, with OS producing higher b* values than SV. This effect became more pronounced under more intensive cooking conditions, as evidenced by the significant interaction among cooking method, temperature, and time. The greater yellowness observed in OS-cooked camel meat is likely attributable to the rapid and uniform volumetric heating associated with ohmic processing, which can accelerate protein denaturation, lipid oxidation, and Maillard browning reactions. These thermal changes promote the formation of yellow- and brown-colored compounds, thereby increasing b* values compared with conventional SV cooking. Furthermore, yellowness (b*) increased with increasing cooking temperature and holding time, irrespective of the cooking method. This trend is likely attributable to greater thermal degradation of meat pigments, enhanced protein denaturation, lipid oxidation, and Maillard browning reactions during prolonged heating, all of which contribute to the formation of yellow- and brown-colored compounds. These findings agree with previous reports [27,48,50], which demonstrated that increasing cooking temperature and duration promote thermally induced physicochemical changes responsible for higher b* values in cooked meat.

The significant differences observed between the two cooking methods for all color attributes can be attributed to the distinct heating mechanisms of the two cooking methods. Unlike conventional SV, the OS method generates heat through the passage of an alternating electric current directly through the meat, resulting in rapid and uniform volumetric heating [51], which leads to swift alterations in the color compounds of the cooked meat [10]. Aydın, Kurt [52] compared ohmic cooking with water bath cooking and reported that ohmic cooking had an important effect on color values. The reason for this was attributed to the occurrence of denaturation and damage to proteins, leading to the leaching of water and mineral elements, and consequently, oxidative reactions. Engchuan, Jittanit [10] also observed changes in color values when compared ohmic cooking with traditional cooking, where ohmic method has reported higher values than those in traditional cooking.

Cooking temperature markedly influenced the color characteristics of camel meat, indicating that thermal intensity is a major determinant of pigment stability and visual appearance. As cooking temperature increased, progressive thermal denaturation of myoglobin, oxidation of the heme iron, and structural modifications of muscle proteins altered light absorption and reflection, resulting in darker, less red meat with greater yellowness, color saturation, hue shift, and overall color change. In addition, elevated temperatures promote Maillard browning and lipid oxidation, further contributing to the formation of yellow- and brown-colored compounds. These findings are consistent with previous reports [10,47,50], which demonstrated that increasing cooking temperature accelerates thermally induced physicochemical changes responsible for the characteristic color modifications observed in cooked meat.

Cooking time also played a significant role in modifying the color characteristics of camel meat, indicating that prolonged thermal exposure intensifies physicochemical changes in muscle pigments and proteins. Extended heating promotes progressive myoglobin denaturation, heme iron oxidation, protein aggregation, and Maillard browning reactions, leading to greater overall color change and a shift toward more yellow and saturated color tones while reducing lightness and redness. These observations are consistent with previous studies [6,27], which reported that prolonged heating enhances thermally induced color changes in meat. Similarly, Yildiz-Turp and Sengun [18] concluded that increasing cooking intensity, through higher temperatures and longer cooking times, accelerates the degradation and transformation of color compounds, thereby altering the visual appearance of cooked meat.

The significant interactions between cooking method and time, and between cooking temperature and time, indicate that the influence of one cooking factor on camel meat color depends on the level of the other. These interactions suggest that color development during cooking is governed by the combined effects of heating intensity and exposure duration rather than by individual factors alone. Variations in the extent of myoglobin denaturation, heme pigment oxidation, protein structural changes, and Maillard browning reactions under different cooking conditions are likely responsible for the observed differences in color attributes. The absence of a significant interaction effect on chroma suggests that color saturation is comparatively less sensitive to these combined processing conditions. These findings are consistent with those of [6], who likewise reported that the combined effects of cooking conditions play a critical role in determining the color characteristics of cooked camel meat.

The significant two- and three-way interactions demonstrate that camel meat color development was governed by the combined effects of cooking method, temperature, and time rather than by the independent influence of each factor. These interactions indicate that the response of individual color attributes to increasing temperature and cooking duration depended on the heating mechanism employed. The observed changes are attributable to progressive myoglobin denaturation, heme iron oxidation, protein structural modifications, and Maillard browning reactions, all of which intensify with increasing thermal severity [47,48]. These effects were more evident in the OS treatment, likely because rapid and uniform volumetric heating accelerates physicochemical reactions responsible for pigment transformation and non-enzymatic browning [50]. These findings highlight the importance of considering cooking method, temperature, and time simultaneously when optimizing thermal processing conditions to achieve desirable color characteristics while minimizing excessive color deterioration in camel meat.

The observed color changes are consistent with the progressive thermal denaturation of myoglobin, oxidation of the heme iron, and the development of Maillard browning products as cooking severity increased. The more uniform appearance of the OS samples is likely attributable to the volumetric heating mechanism of ohmic cooking, which minimizes temperature gradients within the meat and promotes more homogeneous physicochemical changes throughout the sample. In contrast, SV relies solely on conductive heat transfer from the surrounding water bath, which may produce slightly less uniform color development, particularly at shorter cooking times. The observations support the instrumental color results, demonstrating that increasing cooking temperature and time progressively transformed camel meat from a lighter reddish appearance to a darker brown color. Although both cooking methods followed similar color development patterns, OS generally produced a more uniform and slightly darker brown appearance than traditional SV, particularly under the more intensive cooking conditions (80–90 °C and 90–120 min), indicating more homogeneous thermal processing.

The progressive transition from a reddish to a brown color with increasing cooking temperature and time is primarily attributed to thermal denaturation of myoglobin, oxidation of the heme iron, and subsequent pigment transformation [48,50]. In addition, Maillard reactions between reducing sugars and amino compounds contribute to the formation of brown-colored compounds during prolonged heating. The final cooked color is therefore determined by the oxidation state of the heme iron and the extent of myoglobin denaturation, which increase with thermal severity. These observations agree with Hobani et al. [6], who reported that camel meat develops a progressively browner appearance as cooking temperature increases.

#### 4.2.2. The physicochemical properties of camel meat.

The observed differences in the physicochemical characteristics of camel meat between the two cooking methods highlight the influence of the heating mechanism on meat quality. Ohmic-sous vide (OS) cooking generates heat internally through the passage of electrical current across the meat and surrounding medium, producing rapid and uniform volumetric heating. This heating mechanism enhances protein denaturation and structural disruption of muscle fibers, particularly as electrical resistance decreases with increasing temperature, thereby facilitating greater current flow through the meat [53,54]. These structural changes can alter water retention, pH stability, and meat texture through increased moisture migration and the release of soluble components. In contrast, conventional SV relies on indirect heat transfer from a temperature-controlled water bath, resulting in more gradual heating and less pronounced physicochemical modifications [6]. Consequently, OS processing induces greater changes in meat quality attributes than SV, owing to the combined effects of electrical and thermal energy on muscle proteins and tissue structure, which promote moisture and mineral losses and modify the physicochemical properties of the cooked meat [9,18,55].

Cooking temperature was a major determinant of the physicochemical properties of camel meat, reflecting the progressive effects of thermal processing on muscle structure and composition. As thermal intensity increased, greater protein denaturation, collagen solubilization, and disruption of myofibrillar structure promoted moisture migration and the loss of water-soluble minerals, thereby modifying pH, water activity, cooking loss, and meat tenderness. The reduction in shear force at higher temperatures is likely associated with increased connective tissue gelatinization and weakening of the muscle fiber matrix, despite the concurrent increase in moisture loss. These findings are consistent with those of Bozkurt and Icier [8], who demonstrated that ohmic cooking produces distinct quality characteristics compared with conventional heating owing to its rapid and uniform volumetric heat transfer. Similar observations have also been attributed to thermally induced water and mineral losses, which alter the physicochemical properties of cooked meat [9,56].

Similarly, prolonged cooking time markedly influenced the physicochemical characteristics of camel meat, indicating that the duration of thermal exposure is a critical factor governing quality changes. Extended heating promotes progressive protein denaturation, collagen solubilization, and structural degradation of muscle fibers, resulting in greater moisture migration and the release of water-soluble constituents. These changes contribute to increased cooking loss and pH, accompanied by reduced water activity. At the same time, prolonged heating weakens the connective tissue network and disrupts the myofibrillar structure, leading to lower shear force values and improved meat tenderness despite the greater moisture loss. These findings are consistent with those of Hobani et al. [6], who similarly reported that increasing cooking time and temperature during SV and electric oven processing increased pH and cooking loss while reducing water activity in camel meat.

The significant interaction effects indicate that the physicochemical responses of camel meat depended on the combinations of cooking method, temperature, and time. For pH, significant CMD × CTM, CTM × CTI, and CMD × CTM × CTI interactions were observed, whereas the CMD × CTI interaction was not significant. Cooking loss and shear force were significantly affected by all two-way and three-way interactions. Water activity was significantly affected by all two-way interactions, whereas the three-way interaction was not significant. These findings indicate that the main effects of cooking method, temperature, and time should be interpreted together with the responses observed under the individual processing combinations. More broadly, increasing thermal intensity may accelerate protein denaturation, muscle-fiber contraction, collagen solubilization, and moisture migration, thereby modifying pH, cooking loss, water activity, and shear force. Similar observations were reported by [31], who concluded that prolonged exposure to elevated cooking temperatures induces physicochemical changes in red meat through heat-induced modifications of muscle proteins and fiber structure. The increase in pH observed with increasing cooking temperature and duration as presented in the supplementary Figures D-G, particularly during OS processing, reflects the cumulative effects of thermal treatment on muscle biochemistry. Heat-induced denaturation of muscle proteins, degradation of free amino acids, and the formation of basic nitrogenous compounds, such as ammonia, contribute to the increase in pH during cooking. In addition, moisture and mineral losses alter the concentration of ionic constituents within the muscle, further influencing pH values [57,58]. These physicochemical changes become more pronounced as thermal exposure increases and are likely accelerated during OS processing because of its rapid and uniform volumetric heating. Similar observations have been reported by Bozkurt and Icier [8] and other researchers [59], who attributed the increase in meat pH during cooking to heat-induced structural and biochemical modifications of muscle proteins.

The higher pH observed in camel meat cooked by OS compared with conventional SV, particularly under the most severe cooking conditions, can be attributed to differences in the heating mechanism. Unlike SV, which relies on conductive heat transfer from the surrounding water, OS produces rapid and uniform volumetric heating through the application of an electric field. As thermal intensity increases, enhanced protein denaturation, degradation of free amino acids, and the release of water-soluble minerals promote the formation of basic nitrogenous compounds, contributing to a greater increase in pH. The accelerated denaturation of major muscle proteins, including myosin, sarcoplasmic proteins, collagen, and actin, further modifies the physicochemical properties of the meat, with myosin denaturing at lower temperatures than actin [60]. Consequently, OS processing achieves the desired degree of doneness more rapidly than SV because the combined effects of electrical and thermal energy accelerate structural changes within the muscle. The electric field also promotes ionic movement within the cooking medium and, to a lesser extent, through the meat via leakage currents and capacitive effects across the vacuum bag, thereby enhancing heat generation and transfer [49,55]. These observations agree with Bozkurt and Icier [8], who likewise reported higher pH values in ohmically cooked beef than in conventionally cooked samples.

Cooking loss was strongly influenced by cooking method, temperature, and time, indicating that moisture retention in camel meat is highly dependent on the severity of thermal processing. Increasing cooking temperature and prolonging cooking time promote progressive denaturation of myofibrillar proteins, contraction of muscle fibers, and shrinkage of the connective tissue matrix, thereby reducing the water-holding capacity of the muscle and facilitating the release of water and soluble minerals during cooking [61]. These thermally induced structural changes become more pronounced as heating intensity increases, resulting in greater cooking loss. The more substantial changes observed during OS processing are likely attributable to its rapid and uniform volumetric heating, which accelerates protein denaturation and moisture migration compared with conventional SV. These findings are consistent with previous reports [6,62], including the study by Hobani et al. [6], which demonstrated that increasing cooking temperature and duration significantly increase cooking loss in camel meat.

Water activity decreased progressively with increasing cooking temperature and cooking time under both cooking methods. When averaged across all temperature and time treatments, OS-cooked meat exhibited a slightly but significantly higher water activity than SV-cooked meat, with mean values of 0.983 and 0.981, respectively. The decrease in water activity with increasing thermal intensity may be associated with heat-induced protein denaturation, muscle-fiber contraction, and moisture migration during cooking. These findings are consistent with those of Hobani et al. [6], who reported that increasing cooking temperature and duration reduced water activity in camel meat. Similar observations were reported by Dutra et al. [63], who associated the reduction in water activity at higher cooking temperatures with thermally induced alterations in muscle protein structure.

Shear force was also markedly affected by the cooking conditions, reflecting the combined influence of thermal processing on meat tenderness and texture. Increasing cooking temperature and prolonging cooking time reduced shear force, indicating progressive tenderization of the meat. This effect is primarily associated with collagen solubilization, weakening of the connective tissue network, and disruption of the myofibrillar structure, which collectively reduce the force required to shear the cooked meat despite the concurrent increase in cooking loss.

Shear force decreased with increasing cooking temperature and duration, indicating progressive tenderization of camel meat as thermal intensity increased. This reduction is primarily attributed to heat-induced solubilization of collagen, weakening of the perimysium and other connective tissue components, and disruption of the myofibrillar structure, all of which reduce the mechanical resistance of the muscle to shear [33,57,64]. Although moisture loss increased during cooking, the softening of connective tissue and structural breakdown of muscle fibers had a greater influence on tenderness than the hardening effect associated with water loss.

Ohmic-sous vide (OS) processing produced lower shear force values than conventional sous vide (SV), suggesting a greater tenderizing effect. The rapid and uniform volumetric heating generated during OS accelerates protein denaturation and connective tissue degradation, leading to more extensive structural modification of the muscle. In addition, the electric field enhances heat generation within the meat, promoting faster physicochemical changes than those achieved by conductive heating alone [55]. These observations are consistent with the findings of Tian et al. [55], who reported lower shear force in ohmically cooked beef than in water bath-cooked samples due to greater thermal degradation of muscle proteins. The results demonstrate that the combined effects of cooking method, temperature, and time play a critical role in determining the physicochemical and textural quality of cooked camel meat through their influence on moisture retention, protein denaturation, connective tissue integrity, and tenderness.

#### 4.2.3. The sensory evaluation of camel meat.

The sensory evaluation indicated that the cooking method influenced consumer perception of camel meat, particularly with respect to overall acceptability and tenderness, whereas flavor and juiciness were comparatively unaffected. The superior sensory performance of ohmic-sous vide (OS) is likely attributable to its rapid and uniform volumetric heating, which promotes more homogeneous protein denaturation and collagen solubilization throughout the meat. These structural changes enhance tenderness, a key determinant of overall consumer acceptance, without adversely affecting flavor or juiciness. The lower instrumental shear force observed for OS-cooked samples supports the sensory findings, suggesting that improvements in tenderness contributed directly to the higher acceptability scores.

These observations are consistent with previous studies demonstrating the advantages of ohmic cooking over conventional water bath cooking. Tian et al. [55] reported that ohmic cooking produced beef with lower shear force and greater tenderness than water bath cooking, reflecting more extensive modification of muscle proteins and connective tissue. Similar improvements in meat quality following ohmic processing have also been reported by Piette et al. [59] and more recently by [52]. Collectively, these findings suggest that integrating ohmic heating with sous vide technology can enhance the sensory quality of camel meat while preserving other desirable eating attributes. Cooking temperature and time were the primary factors influencing the sensory quality of camel meat, highlighting the importance of thermal intensity in determining consumer acceptability. Increasing thermal exposure improved tenderness and flavor, which can be attributed to progressive collagen solubilization, connective tissue weakening, and the development of desirable flavor compounds through Maillard reactions and lipid-derived volatile formation. Conversely, prolonged or more intense heating reduced juiciness because of greater moisture loss during cooking. Despite this reduction, the improvements in tenderness and flavor appeared to have a greater influence on overall consumer acceptance, indicating that these attributes were the main drivers of eating quality.

The superior sensory performance observed under the more intensive OS cooking conditions is likely associated with the rapid and uniform volumetric heating characteristic of ohmic processing, which promotes more homogeneous structural modifications throughout the meat while reducing temperature gradients. These observations are consistent with previous studies [27,47], as well as the findings of Yildiz-Turp and Sengun [18], who reported that ohmic cooking produces more uniform heating and improves the sensory quality of beef compared with conventional cooking methods. Similar advantages of ohmic processing for enhancing tenderness and overall eating quality have also been reported by Bozkurt and Icier [8], Icier et al. [10], and Tian et al. [55].

The improvement in tenderness with increasing cooking temperature and time is primarily attributed to progressive collagen solubilization, weakening of the connective tissue network, and structural disruption of myofibrillar proteins during thermal processing. These changes reduce the mechanical resistance of the muscle, resulting in a more tender product despite the accompanying moisture loss. In addition, prolonged heating promotes the conversion of collagen to gelatin, further enhancing meat tenderness. These findings are consistent with previous reports [33,61], which demonstrated that heat-induced modifications of connective tissue and muscle proteins are the principal mechanisms responsible for the improved tenderness of cooked meat.

The greater tenderness achieved by ohmic-sous vide (OS) compared with conventional sous vide (SV) is likely attributable to the rapid and uniform volumetric heating generated during ohmic processing, which accelerates protein denaturation, collagen solubilization, and structural weakening of the connective tissue. These physicochemical changes promote a more pronounced tenderizing effect than conductive heating alone. The present findings are consistent with previous studies [59]. Likewise, Tian et al. [55] reported that ohmic cooking produced more tender beef than conventional water bath cooking, with tenderness improving as electric field intensity increased. Hobani et al. [6] also demonstrated that sous vide processing effectively preserves the tenderness of camel meat, highlighting the benefits of controlled low-temperature cooking for maintaining desirable textural properties.

The highest tenderness and flavor ratings were obtained under the most intensive cooking conditions, indicating that prolonged heating at elevated temperatures enhanced the eating quality of camel meat [65]. The improvement in tenderness is primarily associated with collagen solubilization and the progressive weakening of connective tissue, while enhanced flavor development is likely due to the formation of desirable volatile compounds through Maillard reactions and lipid oxidation during cooking. In both cooking methods, vacuum packaging helped preserve the natural flavor of camel meat by minimizing the loss of volatile flavor compounds and reducing oxidative deterioration [6,27]. Furthermore, the rapid and uniform volumetric heating characteristic of OS promotes more homogeneous heat distribution throughout the meat, facilitating consistent flavor development and contributing to improved sensory quality compared with conventional heating methods [66].

The improvement in flavor with increasing cooking temperature and time is likely attributable to the progressive formation of flavor-active compounds during thermal processing. Elevated temperatures and prolonged heating enhance Maillard reactions between reducing sugars and amino acids, as well as lipid oxidation and degradation, generating a wide range of volatile compounds responsible for the characteristic aroma and flavor of cooked meat. In addition, the thermal release and transformation of intramuscular lipids contribute to flavor development by producing lipid-derived volatile compounds that interact with Maillard reaction products, creating the complex flavor profile of cooked camel meat [6]. These mechanisms explain the enhanced flavor perception observed under more intensive cooking conditions.

The decline in juiciness with increasing cooking temperature and time is primarily attributable to progressive moisture loss resulting from protein denaturation, muscle fiber contraction, and reduced water-holding capacity during thermal processing. As heating intensity increases, a greater proportion of intracellular and extracellular water is expelled from the muscle, diminishing the perception of juiciness. Although both cooking methods exhibited this trend, the reduction in juiciness reflects the inevitable trade-off between achieving greater tenderness through prolonged heating and preserving moisture within the meat. These findings are consistent with those of Hobani et al. [6] and Zell et al. [27], who similarly identified cooking temperature and time as the principal factors governing moisture retention and the sensory perception of juiciness in cooked meat.

Under the most intensive cooking conditions, camel meat processed by ohmic-sous (OS) was generally preferred over conventional sous vide (SV), indicating that the benefits of ohmic heating become more evident at higher temperatures and longer cooking times. This preference is likely attributable to the rapid and uniform volumetric heating characteristic of OS, which promotes more homogeneous protein denaturation, connective tissue solubilization, and structural modification throughout the meat. These changes enhance tenderness and overall eating quality while maintaining desirable sensory attributes despite the greater thermal intensity. In addition, the more uniform heat distribution achieved during OS processing minimizes temperature gradients within the meat, contributing to greater consistency in texture and overall consumer acceptance [47,55]. Similar findings have been reported by Zell et al. [27], who concluded that ohmically cooked meat was generally preferred over conventionally cooked beef. The present results also agree with previous studies [9,10,55], supporting the potential of ohmic-sous vide processing to improve the sensory quality and consumer acceptability of cooked camel meat.

The improvement in overall acceptability with increasing cooking temperature and time suggests that thermal intensity plays a key role in enhancing the eating quality of camel meat. Prolonged heating promotes collagen solubilization and gelatin formation, together with the melting of intramuscular fat, resulting in a more tender texture and enhanced flavor development, both of which contribute positively to consumer acceptance [67,68]. The significant interaction between cooking temperature and time for tenderness and flavor further indicates that these sensory attributes are jointly governed by the severity and duration of heat treatment. In contrast, the absence of significant interactions involving cooking method suggests that the effects of temperature and time on overall acceptance and juiciness were largely independent of the heating technique employed, indicating comparable sensory responses to thermal processing under both SV and OS conditions.

## 5. Conclusion

A novel OS cooking system was developed and compared with traditional SV cooking to evaluate its effects on the physicochemical, color, textural, and sensory properties of camel meat. Significant differences were observed between the two cooking methods. OS outperformed traditional SV due to its ability to generate heat through electric current, maintain a constant cooking temperature, and induce favorable protein modifications during extended cooking. Optimal results were achieved at 90 °C for 120 min, producing a more homogeneous and tender product. Additionally, vacuum packaging contributed to improved meat quality retention. The combined effects of uniform heating and electric current resulted in greater tenderness and higher consumer acceptability compared to the conventional SV method. Future studies should investigate the electrical-current and electric-field pathways within the OS system, determine the electrical resistance and dielectric properties of the cooking pouches, and characterize current distribution and internal temperature profiles within the meat samples. Additional research should also evaluate energy consumption, microbial safety, protein and structural changes, scale-up feasibility, and the performance of the OS system in other meat and food products before industrial application is considered.

## Supporting information

S1 File Figures (A-K) showing impact of cooking method, temperature, and time on physicochemical and sensory attributes of camel meat are provided for in depth understanding of the interrelations between the studied variables. (DOCX)
