# Supplementary material for: Innovative ohmic-sous vide cooking technique for enhancing physicochemical, textural, and sensory attributes of Arabian camel (Camelus dromedarius) meat
Source: PLoS One. 2026 Nov 3;21(11):e0359223. doi: 10.1371/journal.pone.0359223 (PMC13730367; doi:10.1371/journal.pone.0359223)
Supplement: Supplementary file 1 [file pone.0359223.s001.docx]

^a,b,c^ Columns bearing different letters are significantly different at p<0.05.

Lightness

| Time (min) | Sous vide cooking 70°C | Sous vide cooking 80°C | Sous vide cooking 90°C | Ohmic-sous vide cooking 70°C | Ohmic-sous vide cooking 80°C | Ohmic-sous vide cooking 90°C |
| --- | --- | --- | --- | --- | --- | --- |
| 30 | 54.736 | 44.756 | 39.25 | 50.228 | 45.995 | 42.4108 |
| 60 | 49.358 | 38.476 | 36.476 | 44.314 | 42 | 39.694 |
| 90 | 42.188 | 36.236 | 33.29 | 38.046 | 36.25 | 33.876 |
| 120 | 36.922 | 33.784 | 30.912 | 34.034 | 32.192 | 30.288 |

**Figure A.** Effects of sous vide and ohmic-sous vide cooking on the camel meat lightness(L*) at different cooking temperatures and times.

Redness

| Time (min) | Sous vide cooking 70°C | Sous vide cooking 80°C | Sous vide cooking 90°C | Ohmic-sous vide cooking 70°C | Ohmic-sous vide cooking 80°C | Ohmic-sous vide cooking 90°C |
| --- | --- | --- | --- | --- | --- | --- |
| 30 | 13.668 | 11.262 | 9.94 | 11.122 | 9.4 | 8.466 |
| 60 | 11.506 | 9.694 | 7.818 | 10.314 | 8.252 | 7.35 |
| 90 | 10.12 | 8.65 | 6.662 | 8.868 | 7.166 | 6.236 |
| 120 | 9.346 | 7.208 | 6.036 | 7.86 | 6.578 | 5.648 |

^a,b,c^ Columns bearing different letters are significantly different at p<0.05.

**Figure B.** Effects of sous vide and ohmic-sous vide cooking on the camel meat redness(a*) at different cooking temperatures and times.

^a,b,c^ Columns bearing different letters are significantly different at p<0.05.

Yellowness

| Time (min) | Sous vide cooking 70°C | Sous vide cooking 80°C | Sous vide cooking 90°C | Ohmic-sous vide cooking 70°C | Ohmic-sous vide cooking 80°C | Ohmic-sous vide cooking 90°C |
| --- | --- | --- | --- | --- | --- | --- |
| 30 | 9.192 | 10.372 | 12.192 | 13.322 | 15.78 | 18.218 |
| 60 | 11.568 | 12.512 | 13.494 | 15.282 | 16.166 | 19.418 |
| 90 | 13.098 | 13.77 | 14.952 | 16.756 | 17.6325 | 19.718 |
| 120 | 14.3225 | 15.532 | 16.234 | 17.884 | 19.028 | 20.308 |

**Figure C.** Effects of sous vide and ohmic-sous vide cooking on the camel meat yellowness(b*) at different cooking temperatures and times.

pH

| Time (min) | Sous vide cooking 70°C | Sous vide cooking 80°C | Sous vide cooking 90°C | Ohmic-sous vide cooking 70°C | Ohmic-sous vide cooking 80°C | Ohmic-sous vide cooking 90°C |
| --- | --- | --- | --- | --- | --- | --- |
| 30 | 5.872 | 5.997 | 6.09 | 5.95 | 6.006 | 6.07267 |
| 60 | 6.01 | 6.07167 | 6.16 | 6.088 | 6.136 | 6.19933 |
| 90 | 6.086 | 6.12267 | 6.232 | 6.138 | 6.17 | 6.27333 |
| 120 | 6.134 | 6.24233 | 6.31 | 6.188 | 6.244 | 6.36733 |

^a,b,c^ Columns bearing different letters are significantly different at p<0.05.

**Figure D.** Effects of sous vide and ohmic-sous vide cooking on camel meat pH at different cooking temperatures and times.

^a,b,c^ Columns bearing different letters are significantly different at p<0.05.

Cooking Loss (%)

| Time (min) | Sous vide cooking 70°C | Sous vide cooking 80°C | Sous vide cooking 90°C | Ohmic-sous vide cooking 70°C | Ohmic-sous vide cooking 80°C | Ohmic-sous vide cooking 90°C |
| --- | --- | --- | --- | --- | --- | --- |
| 30 | 21.9294 | 26.8239 | 30.6816 | 23.3256 | 33.7587 | 41.1708 |
| 60 | 26.7018 | 34.7754 | 37.1478 | 34.9604 | 39.1148 | 44.4349 |
| 90 | 33.0411 | 37.3121 | 41.2426 | 37.536 | 41.471 | 47.0665 |
| 120 | 37.0237 | 39.8095 | 44.4795 | 41.2141 | 44.4843 | 52.6879 |

**Figure E.** Effects of sous vide and ohmic-sous vide cooking on camel meat cooking loss at different cooking temperatures and times.

Water activity (aw)

| Time (min) | Sous vide cooking 70°C | Sous vide cooking 80°C | Sous vide cooking 90°C | Ohmic-sous vide cooking 70°C | Ohmic-sous vide cooking 80°C | Ohmic-sous vide cooking 90°C |
| --- | --- | --- | --- | --- | --- | --- |
| 30 | 0.995 | 0.993 | 0.99 | 0.9932 | 0.987 | 0.9848 |
| 60 | 0.993 | 0.99 | 0.985 | 0.986 | 0.9848 | 0.983 |
| 90 | 0.991 | 0.989 | 0.983 | 0.9828 | 0.9812 | 0.9786 |
| 120 | 0.989 | 0.987 | 0.981 | 0.9798 | 0.9772 | 0.9732 |

^a,b,c^ Columns bearing different letters are significantly different at p<0.05.

**Figure F.** Effects of sous vide and ohmic-sous vide cooking on camel meat water activity (a_w_) at different cooking temperatures and times.

^a,b,c^ Columns bearing different letters are significantly different at p<0.05.

Shearing force (N)

| Time (min) | Sous vide cooking 70°C | Sous vide cooking 80°C | Sous vide cooking 90°C | Ohmic-sous vide cooking 70°C | Ohmic-sous vide cooking 80°C | Ohmic-sous vide cooking 90°C |
| --- | --- | --- | --- | --- | --- | --- |
| 30 | 50.1654 | 38.7711 | 32.9112 | 41.1168 | 33.6148 | 27.0248 |
| 60 | 40.9722 | 32.8117 | 23.0969 | 34.0852 | 28.2482 | 17.7823 |
| 90 | 34.4459 | 27.9876 | 18.0493 | 30.1004 | 20.4883 | 13.0165 |
| 120 | 26.2083 | 18.1603 | 14.8788 | 21.6248 | 14.7423 | 9.92917 |

**Figure G.** Effects of sous vide and ohmic-sous vide cooking on camel meat shearing force at different cooking temperatures and times.

Tenderness

| Time (min) | Sous vide cooking 70°C | Sous vide cooking 80°C | Sous vide cooking 90°C | Ohmic-sous vide cooking 70°C | Ohmic-sous vide cooking 80°C | Ohmic-sous vide cooking 90°C |
| --- | --- | --- | --- | --- | --- | --- |
| 30 | 2 | 3 | 3.3 | 2.22222 | 3.2 | 3.8 |
| 60 | 3.55556 | 4.7 | 5.3 | 3.55556 | 5 | 5.8 |
| 90 | 4.22222 | 5.9 | 6.9 | 4.66667 | 5.9 | 6.9 |
| 120 | 5 | 6.7 | 7.9 | 5.55556 | 6.6 | 7.9 |

^a,b,c^ Columns bearing different letters are significantly different at p<0.05.

**Figure H.** Effects of sous vide and ohmic-sous vide cooking on camel meat tenderness at different cooking temperatures and times.

^a^^,b,c^ Columns bearing different letters are significantly different at p<0.05.

Flavor

| Time (min) | Sous vide cooking 70°C | Sous vide cooking 80°C | Sous vide cooking 90°C | Ohmic-sous vide cooking 70°C | Ohmic-sous vide cooking 80°C | Ohmic-sous vide cooking 90°C |
| --- | --- | --- | --- | --- | --- | --- |
| 30 | 2.33333 | 3.3 | 4.6 | 2.6 | 3.3 | 4.6 |
| 60 | 3.5 | 4.6 | 5.9 | 3.7 | 4.7 | 5.5 |
| 90 | 4.5 | 5.7 | 6.8 | 4.7 | 5.5 | 6.4 |
| 120 | 5.6 | 6.4 | 7.8 | 5.5 | 6.6 | 7.6 |

**Figure I.** Effects of sous vide and ohmic-sous vide cooking on camel meat flavor at different cooking temperatures and times.

Juiciness

| Time (min) | Sous vide cooking 70°C | Sous vide cooking 80°C | Sous vide cooking 90°C | Ohmic-sous vide cooking 70°C | Ohmic-sous vide cooking 80°C | Ohmic-sous vide cooking 90°C |
| --- | --- | --- | --- | --- | --- | --- |
| 30 | 7.2 | 5.9 | 5 | 7 | 6 | 5.5 |
| 60 | 6.1 | 4.9 | 4.1 | 6 | 5 | 4.1 |
| 90 | 5.1 | 4.3 | 3.1 | 5 | 4.2 | 3.5 |
| 120 | 4 | 2.9 | 2.3 | 3.9 | 3.3 | 2.9 |

^a,b,c^ Columns bearing different letters are significantly different at p<0.05

**Figure J.** Effects of sous vide and ohmic-sous vide cooking on camel meat juiciness at different cooking temperatures and times.

General Acceptance

| Time (min) | Sous vide cooking 70°C | Sous vide cooking 80°C | Sous vide cooking 90°C | Ohmic-sous vide cooking 70°C | Ohmic-sous vide cooking 80°C | Ohmic-sous vide cooking 90°C |
| --- | --- | --- | --- | --- | --- | --- |
| 30 | 2.4 | 3.6 | 4.3 | 2.6 | 3.6 | 4.9 |
| 60 | 3.2 | 4.3 | 5.5 | 3.6 | 5 | 6.1 |
| 90 | 4.5 | 5.6 | 6.5 | 4.9 | 5.9 | 6.8 |
| 120 | 5.5 | 6.3 | 7.9 | 5.6 | 7 | 7.9 |

^a,b,c^ Columns bearing different letters are significantly different at p<0.05

**Figure K.** Effects of sous vide and ohmic-sous vide cooking on camel meat general acceptance at different cooking temperatures and times.
